# LXR Agonists as Therapeutic Agents for Myelin Repair: Exploring the Molecular Mechanisms of Oligodendrocyte Remyelination and Cholesterol Synthesis

**DOI:** 10.1002/alz.091722

**Published:** 2025-01-09

**Authors:** Sampada P Tamhankar, Ian Steinke, Meenakshi Singh, Raj Amin

**Affiliations:** ^1^ Auburn University, Auburn, AL USA

## Abstract

**Background:**

Alzheimer’s disease (AD) is characterized by hallmark amyloid plaques and neurofibrillary tangles as well as by a significant loss of myelin in the cerebral cortex and other brain regions, which contributes to neurodegeneration and cognitive decline. Remyelination, of the myelin sheath by oligodendrocytes, is a process that may be impaired in neurodegenerative diseases. Depending on the severity of the disease, there occurs loss or partial damage of the myelin sheath surrounding the neuron leading to memory deficits. Liver X Receptors (LXRs), as regulators of cholesterol homeostasis, present an intriguing target for therapeutic intervention, as cholesterol is a vital component of myelin. The proposed research seeks to investigate the potential of LXR agonists to stimulate myelin production in oligodendrocytes, thereby promoting remyelination in the AD brain. This study will assess the capacity of LXR‐induced remyelination to alleviate cognitive deficits associated with demyelination in AD pathology.

**Method:**

Wild type and 5XFAD mice were used for in‐vivo and MO3.13‐ human hybrid cell lines that express phenotypic characteristics of primary oligodendrocyte were used for in vitro studies. A Potent LXR agonist (GW3965) was used to study the remyelination properties The cell lysate was then resolved using SDS PAGE – western blot analysis.

**Result:**

5XFAD mice show comparatively lower expression of pre‐ and mature oligodendrocyte biomarkers compared to wild types. As per the literature, M03.13 has genes for myelination but does not express any associated protein. We have observed that the MO3.13 cell line expresses the pre‐myelination markers but lacks the mature myelination marker. Surprisingly, treatment with GW3965 induces myelination markers expression.

**Conclusion:**

The current study provides evidence that LXR agonists can be used to enhance the myelination properties within the oligodendrocyte. Further work will focus on the application of LXR agonists in Frontal temporal dementia and ALS‐related neurodegenerative disease.

